# Omega-3 PUFA Responders and Non-Responders and the Prevention of Lipid Dysmetabolism and Related Diseases

**DOI:** 10.3390/nu12051363

**Published:** 2020-05-10

**Authors:** Simona Serini, Gabriella Calviello

**Affiliations:** Department of Translational Medicine and Surgery, Division of General Pathology, School of Medicine, Università Cattolica del S. Cuore, L.go F. Vito, 1 00168 Rome, Italy; simona.serini@unicatt.it

**Keywords:** dietary supplement, dysmetabolism, omega-3 index, omega-6/omega-3 PUFA ratio, responders, non-responders

## Abstract

The long-chain omega-3 polyunsaturated fatty acids (LC-omega-3 PUFAs) eicosapentaenoic acid and docosahexaenoic acid are the most popular dietary supplements recommended for the prevention/management of lipid dysmetabolisms and related diseases. However, remarkable inconsistencies exist among the outcomes of the human intervention studies in this field, which contrast with the impressive homogeneity of positive results of most of the preclinical studies. In the present review, we will firstly examine a series of factors—such as background diet composition, gut microbiota and genetic/epigenetic variants, which may lie beneath these inconsistencies. Moreover, we will discuss the recent advance in the knowledge of possible specific biomarkers (genetic-, epigenetic- and microbiota-related) that are being investigated with the goal to apply them in a personalized supplementation with omega-3 PUFAs. We will also consider the possibility of using already available parameters (Omega-3 index, Omega-6 PUFA/Omega-3 PUFA ratio) able to predict the individual responsiveness to these fatty acids and will discuss the optimal timing for their use. Finally, we will critically examine the results of those human studies that have already adopted the distinction of the subjects into omega-3 PUFA responders and non-responders and will discuss the advantage of using such an approach.

## 1. Introduction

The long-chain omega-3 polyunsaturated fatty acids (LC-omega-3 PUFAs) eicosapentaenoic acid (EPA, 20:5 n-3) and docosahexaenoic acid (DHA, 22:6 n-3) are the most popular over-the-counter dietary supplements recommended for the prevention and management of lipid dysmetabolisms and related cardiovascular diseases (CVDs) [1,2]. Compared to other widely used nutritional supplements, such as polyphenols or carotenoids, these fatty acids, besides being normal components of our diet (especially found in fish and seafood), can be endogenously produced by sequential elongation and desaturation steps from their precursor, the shorter-chain and essential nutrient α-linolenic acid (ALA, 18:3 n-3), widely found in foods of vegetable origin [3]. Moreover, they are structural constituents of our cell membranes, and can also be metabolically converted into a series of bioactive compounds.

One intriguing point regarding these LC-omega-3 PUFAs is that, in spite of their large use as dietary supplements, especially for the prevention of blood lipid dysmetabolism and CVDs, remarkable inconsistencies exist among the outcomes of human intervention studies investigating their ability to reduce the development and progression of these disorders [2,4]. As a result, most of the meta-analyses recently published in the CVD research field conclude that, overall, they do not induce statistically significant health effects, and that their use for the management of blood lipid dysmetabolism and CVDs should be further reconsidered [2]. Thus, clarity regarding whether supplementation or an increased dietary intake of LC-omega-3 PUFAs may be useful for the prevention of these pathologies is still lacking. Similar conclusions have also been drawn by meta-analyses of the human studies focusing on the preventive and adjuvant therapeutic usage of LC-omega-3 PUFAs against the main human cancers [5,6,7]. Yet, the high degree of inconsistency observed between human studies, especially in some fields of omega-3 PUFA research, including CVDs and cancer, contrasts with the impressive homogeneity of positive results obtained in almost all the preclinical studies performed in animals. However, we cannot forget that the experimental conditions used in the animal studies (i.e., background diet and total amount of omega-3 and omega-6 PUFAs ingested; genetic background; environmental conditions such as temperature, humidity, light, etc. used during the experiments) are more homogeneous and strictly controlled as compared to those of the human studies. A factor that should not be ignored when considering the inconsistencies in the outcomes of different meta-analyses is also the extremely variable bioavailability that can be obtained when different forms of these fatty acids (free fatty acids—FFAs—or esterified fatty acids) are ingested in diets or supplements. Moreover, recently, it has become increasingly clear that, within a human population, different individuals may show a diverse degree of response to LC-omega-3 PUFA dietary supplementations [8]. Altogether, these factors may concur to the lack of statistically significant effects that are often reported in human studies or meta-analyses evaluating the beneficial activity of LC-omega-3 PUFAs, especially in the CVD and oncologic research fields. Furthermore, the responses of different populations to these fatty acids may vary considerably, thus explaining the inconsistency found between studies performed on populations with varying ethnic backgrounds or living in different countries. Thus, some human studies have started to use the useful strategy of sub-dividing the participants into omega-3 PUFA responders (“Rs”) and omega-3 PUFA non-responders (“NRs”), i.e., individuals that show the beneficial effects induced by LC-omega-3 PUFAs and those that do not. It has been suggested that heterogeneity in response to treatment with omega-3 PUFAs may be explained by genetic [5,9,10,11] or epigenetic [12] variability between individuals. Specifically, it has been hypothesized that the epigenetic make-up during the developmental age, being influenced by the environment and the personal history of each individual, may modify the expression of a variety of genes. Variations of genes involved in the metabolism of omega-3 PUFAs—or the alterations in their epigenetic regulation—have drawn particular attention, even though it cannot be excluded that other factors may play a role in making an individual an “R” or an “NR” to omega-3 PUFA supplementation.

## 2. Objectives of the Review

**Figure 1:**
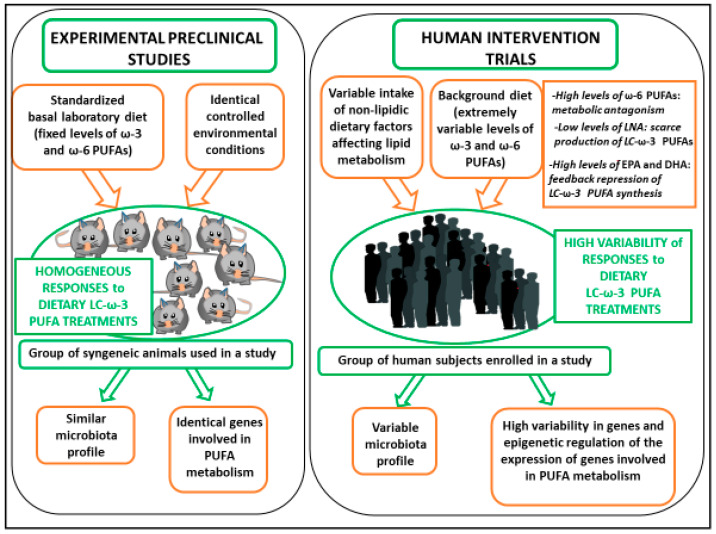
Reasons for the inconsistencies observed in the outcomes of the human LC-ω-3 PUFA intervention trials as compared to experimental preclinical studies.

The first aim of this review is to examine a series of factors (such as the composition of the background diet, the gut microbiota, and the genetic/epigenetic variants) which may lie beneath the inconsistency observed in the intervention human studies vs. the preclinical studies performed with omega-3 PUFAs in the field of lipid dysmetabolism and CVDs (see also Figure 1).

Since it is becoming increasingly clear that, based on these factors, the individuals in a given human population may respond differently to a supplementation containing these fatty acids, new approaches are starting to be used in designing studies with omega-3 PUFAs as well as in analyzing their results. Therefore, the second aim of this review is to examine critically the results of those human studies that have so far used the useful strategy of sub-dividing the participants into omega-3 PUFA “Rs” and “NRs”. The advantage of using such an approach will be also discussed.

## 3. Methodology

A systematic literature search of the PubMed database was conducted from September 2019 to February 2020 to identify published peer-reviewed original articles regarding the effects of omega-3 PUFAs on dysmetabolisms in human studies. The key words used for the search of titles and abstracts were: “Omega-3 PUFA” or “Docosahaxaenoic acid” or “Eicosapentaenoic acid” or “Fish oil” and “supplementation” and “dysmetabolism” or “lipoproteins” or “hypertriglyceridemia” or “cardiovascular” and “human studies” and “responders” and “microbiota” and “genetic” and “polymorphism” and “epigenetics”. We identified full-text articles written in English. The papers were chosen without restriction of time.

## 4. Discussion

### 4.1. Human vs. Preclinical Studies, Reasons for the Inconsistencies

#### 4.1.1. Influence of Background Diets

A first important point that can help to understand why the results of the human studies are usually more inconsistent as compared to those obtained in the preclinical studies is that the laboratory animal diets are designed to ensure precise and optimal basal intakes of essential components of mammal diets such as omega-6 and omega-3 PUFAs. In contrast, a large variability exists in the basal dietary intake of these fatty acids between the individuals within a human population [13,14]. Furthermore, in the studies specifically investigating the effects of dietary omega-3 PUFAs in animals, controlled doses of these fatty acids are given as an oral supplement (often by gavage) in addition to the basal diets or are added to the basal diet itself. These procedures have also allowed the discovery of how different basal laboratory diets with either low- or high-fat levels may influence the effects induced by a further supplementation with omega-3 PUFAs [15,16,17]. This suggests that it would be also extremely important to evaluate the actual background dietary intake of these fatty acids as well as those of other classes of lipids in each of the participants in intervention clinical trials, and, possibly, try to analyze the results by stratifying them according to these variables. In the following paragraphs we will focus on several of these different dietary variables and will consider also the strategies that have been used so far to take them into proper account.

##### Influence of the Background Levels of Dietary Omega-3 PUFAs

It has been largely discussed how difficult is to evaluate the background level of LC-omega-3 PUFA intake by using questionnaires to obtain the information [18]. It has been suggested that the direct evaluation of the LC-omega-3 PUFA levels in each subjects’ cells/tissues could be more helpful [18]. In fact, the ability of LC-omega-3 PUFAs to induce health effects has been usually related to their level in tissues, otherwise known as the omega-3 tissue status. Particularly, the level of omega-3 fatty acids in the erythrocyte membranes (Omega-3 index) has often been proposed as a useful marker directly related to the omega-3 PUFA dietary intake, given its feasibility [19]. Since the Omega-3 index depends also on individual metabolic ability to incorporate these fatty acids in cell membranes, it represents a more complete parameter compared to the level of dietary intake.

It is known that remarkable differences exist also in the basal dietary intake of omega-3 PUFAs between different populations, and these differences should be taken into account in the meta-analyses examining the outcomes of a considerable number of studies performed on different populations that often show extremely variable dietary habits.

##### Influence of the Background Levels of Dietary Omega-6 PUFAs

Furthermore, the large variability in the daily intake of omega-6 PUFAs, and in particular that of linoleic acid (LA, 18:2 ω-6), may also contribute to explaining why extremely different levels of LC-omega-3 PUFAs can be found in the tissues of different individuals, in spite of a comparable amount of dietary omega-3 PUFAs that have been ingested. In fact, as already mentioned, EPA and DHA incorporated in tissues do not derive only from the diet, but they may also be endogenously synthesized through a pathway starting from ALA, which is their metabolic precursor. Sequential enzymatic steps of elongation and desaturation allow ALA to be converted into EPA and, eventually, to DHA. However, this synthetic pathway is negatively influenced by the presence of LA, which competes with ALA for the same desaturation enzyme (Δ-6 desaturase) to be converted into the LC-omega-6 PUFA arachidonic acid (AA, 20:4 ω-6). Even though ALA shows a higher affinity than LA for the Δ-6 desaturase, the latter is the most represented PUFA in the Western diet, thus limiting the endogenous production of EPA and DHA. Moreover, it should be underlined that AA is the precursor of a series of oxygenated derivatives, most of which have been found to possess health-threatening activities, being able to induce pro-inflammatory, pro-thrombotic, and pro-carcinogenic effects. Thus, it has been demonstrated that the health effects of LC-omega-3 PUFAs can be antagonized by the simultaneous incorporation of omega-6 PUFAs in tissues [20]. Therefore, it has also been often recommended that, as a starting point for the human studies, it should be essential to evaluate the n-6 PUFA/n-3 PUFA ratio of the human subject red blood cells, since this parameter is directly able to indicate the proportions at which the two different classes (omega-6 and omega-3) of PUFAs are present in tissues [21]. Not differently from the Omega-3 Index, this parameter is also subject to wide variations among people and populations. Finally, it cannot be ignored that the level of intake of EPA and DHA themselves in the background diet may influence the biosynthetic pathway going from ALA to EPA and DHA. As the final products of this pathway, their abundance reasonably induces a feedback repression of the synthetic enzymes [22].

##### Influence of Other Non-Lipidic Components in the Background Diet

The levels of other dietary non-lipidic components may also affect the individual effectiveness of a dietary treatment with omega-3 PUFAs. For instance, B vitamin status was recently involved as a factor that may deeply influence the efficacy of the omega-3 PUFA action in different subjects [23]. In particular, it has been observed that the baseline levels of plasma total homocysteine, which is considered a marker of vitamin B status, is able to modify the effect that a dietary supplementation with omega-3 PUFAs may have on cognitive performance in patients affected by moderate forms of Alzheimer’s disease [23].

#### 4.1.2. Influence of Gut Microbiota

Microbiota is a dynamic and complex community of microorganisms colonizing our body. The huge number of microorganisms, together with their genes and metabolic products, substantially affects the homeostasis of the host [24]. In particular, the microbiota residing in the gut has the potential to deeply affect the host health and immune response [25]. An optimal interspecies balance exists, and this has been related to a healthy state. However, host genotype and a series of environmental factors, including poor colonization, antibiotics therapy, lifestyle, age, and an unhealthy diet can perturb this equilibrium and induce microbiota dysbiosis, that, in turn, has been related to the development of a series of human diseases [25,26]. Due to the influence that many factors may have on it, the microbiota profile may differ substantially between different individuals. However, by using metagenomic approaches, it was possible to demonstrate that healthy subjects show a microbiota profile characterized by the prevalence of few commensal microbial species, while others are less prevalent [27].

Two kinds of relationships may exist between the gut microbiota and the dietary interventions with omega-3 PUFAs. On one hand, it has been observed that omega-3 PUFAs may exert their protective activity through the induction of healthy modifications in the gut microbiota composition and, on the other hand, an established microbiota could also influence the efficacy of omega-3 PUFAs [28]. In fact, these fatty acids may influence and antagonize several potentially harmful environmental factors that, in turn, are able to affect the gut microbiota composition and balance causing dysbiosis [29]. Among them, the beneficial influence of omega-3 PUFAs on dysbiosis caused by exposure to microorganisms, antibiotic use [30] or pharmacological therapies, as well as the consumption of a high fat diet [31] have been recently considered. Moreover, since the genetic background and multiple environmental variables may shape the gut microbiota composition [32], different individuals may show a diverse microbiota profile, under the effects of extremely variable microbiota-affecting stimuli. In this contest, omega-3 PUFA supplementation may represent only one among a series of stimuli able to affect the microbiota, and, in the same individuals, it can be counterbalanced by stimuli having opposite effects or be synergistically enforced by stimuli showing similar effects. In this quite complicated network, the animal studies make things easier, since they consider a single colony of mice or rats that are fed since weaning an identical background diet and are always exposed to identical environmental conditions. These strictly controlled experimental conditions may allow a better identification of the effects that an increased intake of omega-3 PUFAs has on the composition of microbiota, and, in turn, the possible influences that these modifications may have on the healthy effect induced by omega-3 PUFAs. The results of a series of pre-clinical animal studies published so far have demonstrated that omega-3 PUFA supplementation may positively modify gut microbiota composition and prevent dysbiosis [28,31,33]. On the contrary, the outcomes of the human studies often appear contrasting or inconclusive [28,34]. In particular, one recent finding by Djuric et al. [34] focuses on the possibility that the individual microbiota profile may influence the efficacy of a supplementation with omega-3 PUFAs. These authors treated healthy subjects with a personalized omega-3 PUFA supplementation (ranging from 2 to 10 g/day) based on blood fatty acid response. They evaluated the dose needed to achieve a specific EPA/AA ratio that, according to a mathematical model, was able to predict a 50% reduction in colonic Prostaglandin E2 (PGE2). This is worth noticing, since the level of PGE2 in colonic mucosa represents a marker of inflammation strictly related to colonic mucosa carcinogenesis [35]. The authors found that a subgroup of individuals showing high abundance of the Bacteroides *Prevotella*, both in colonic mucosa biopsy and brush-border samples, resulted resistant to the omega-3 PUFA anti-inflammatory effects (decrease in colonic PGE2). Even though these results have not been obtained in relation to lipid dysmetabolism and related CVDs, they appear to be particularly stimulating, since high *Prevotella* abundance had been previously observed in association to inflammatory conditions and even to high blood cholesterol [36,37]. This observation strongly suggests that the individual gut microbiota pattern may represent an additional factor able to deeply influence the efficacy of an omega-3 PUFA supplementation in reverting an inflammatory status or a condition of dyslipidemia and associated diseases.

#### 4.1.3. Influence of Genetic and Epigenetic Variations

##### Variation of Genes Involved in Omega-3 PUFA Metabolism

In the pre-clinical studies, syngeneic animals are used. This means that all the animals in a study express identical variants of the genes encoding the enzymes involved in the metabolic pathways converting short-chain omega-3 PUFAs into LC-omega-3 PUFAs, or these fatty acids into a series of bioactive lipidic derivatives. Therefore, since all the animals are fed strictly controlled diets, these metabolic activities are supposed to function at a comparable level in all the animals. On the other hand, in different individuals within a human population, large variability exists in genes encoding for the desaturases and elongases of the PUFA synthetic pathways [38]. This variability may contribute to explain the inter-individual (and inter-population) differences existing in the cell/tissue capacity of incorporating EPA and DHA. In fact, depending on the gene variants possessed, the subjects may have a high propensity or a very scarce attitude to synthesize LC-omega-3 PUFAs from ALA and accumulate them in red blood cells/lipids or in tissues. It has been suggested that, as a result, these different genotypes could be associated respectively with low or high risk for inflammation and some diet-related chronic diseases showing inflammation as the key pathogenic step, including CVDs [39]. However, it has also been suggested that personalized dietary fatty acid intake could have the potential to modify the relationship between these gene variants and the levels of these fatty acids that can be accumulated in cells and tissues [39].

Moreover, the health effects of LC-omega-3 PUFAs have been related to the intrinsic activities of these fatty acids, as well as to those of their oxygenated derivatives (prostaglandins, thromboxanes, leukotrienes, resolvins, protectins). In fact, on the one hand, these fatty acids may act in part by themselves, thanks to their ability to specifically bind to specific receptors [40,41] or by modulating the physico-chemical lipid microenvironments (i.e., rafts) of plasma membranes, and the activity of a multiplicity of molecular factors/pathways residing in those micro-regions [42,43]. However, a substantial role seems to be played also by the resolvins and protectins [44], which have been demonstrated to be even more bioactive than their precursors, showing powerful inhibitory and pro-resolving roles in the inflammatory processes [45]. It has been hypothesized that the presence of different variants of genes codifying for enzymes involved in these metabolic conversions of LC-omega-3 PUFAs in different subjects within a human population could help to explain the discrepancies among different studies focusing on the health-promoting effects of these fatty acids [46].

##### Epigenetic Changes Affecting the Expression of Genes Involved in Omega-3 PUFA Metabolism

Unlike laboratory animals that are exposed to strictly controlled environmental conditions at all stages of their life and during the experiments, everyone in a human population has a personal history and is subject to environmental factors that can vary dramatically from individual to individual. Under the influence of casual environmental factors (such as insufficient or excessive nutrition), and especially during the early plastic phases of our life, epigenetic modifications may take place and induce alterations of gene expression [47]. Particularly, it has been observed that epigenetic changes may regulate the transcription of genes codifying for the enzymes involved in omega-3 PUFA metabolism [48,49]. Since the epigenetic modulation of gene expression is related to variable environmental influences, it may variably shape the individual ability to metabolize and accumulate LC-omega-3 PUFAs in tissues.

##### Genetic and Epigenetic Regulation of Enzymes Involved in Omega-3 PUFA Metabolism: Outcomes of Human Studies

Several reports have supported the hypothesis that variants of genes involved in the metabolism of omega-3 PUFAs as well as alterations in the epigenetic regulation of these genes may help to explain why the beneficial activity of these fatty acids can be observed only in some individuals. However, the detailed analysis of the available studies in this field is beyond the aim of this review, and a comprehensive review of the literature was recently published on this topic [8]. In particular, it covered the genetic and epigenetic variations in genes (including FADS1, FADS2, ELOVL5, and ELOVL2) of the LC-omega-3 PUFA endogenous synthetic pathway [8] that had been directly associated to serum and tissue changes in the levels of these fatty acids. However, it is worth mentioning here the results of a more recent report by the same group of authors as that review. In this report, they further investigated the genetics of the rate-limiting steps in the metabolic process catalyzed by the desaturases FADS1 and FADS2 [46] by examining 44 different tissues from the Genotype-Tissue Expression Project. They evaluated the impact that genetic single nucleotide polymorphisms (SNPs) in and near FADS gene cluster could have on the expression of FADS1 and FADS2. In particular, they observed that more than half of the tissues examined contained significant quantitative trait loci (eQTLs, i.e., loci that explain variance in expression traits) for either FADS1 or FADS2, and that six tissues (i.e., artery, esophagus, heart, muscle, nerve, and thyroid) showed significant eQTLs associated with both FADS1 and FADS2. What is most intriguing to us is that the eQTLs identified in the six tissues were associated always with changes in the expression of the two FADS genes going in opposite directions. This raises new questions on how the opposite regulation of FADS1 and FADS2 expression may influence LC-omega-3 PUFA metabolism and enrichment in specific tissues. This is crucial, since the beneficial action of these fatty acids may be related to their concentrations. For instance, in the central nervous system, LC-omega-3 PUFAs, and specifically DHA, represent main constituents, and depending on their concentrations, may protect from the risk of inflammation-related diseases, or even increase that risk [50,51,52]. Similarly, high levels of LC-omega-3 PUFA incorporation in prostate tissue has been hypothesized to be associated to an increased risk of cancer, while lower levels are believed to exert a protective anticancer activity in that district [53].

The results obtained recently in a preclinical study by Gromovsky et al. [54] are also worth mentioning. They unequivocally succeed in demonstrating the role of FADS1 as a regulator of inflammation and resolution. The authors used hyperlipidemic mice [for a mutation in the low-density lipoprotein (LDL) receptor] treated with antisense oligonucleotide targeting the selective knockdown of FADS1. The complete loss of activity of this enzyme caused deep alterations in the levels of LC-omega-3 and -6 PUFAs, as well as in those of their pro-resolving or proinflammatory metabolic products in the tissues investigated. Moreover, the animals displayed hepatic inflammation and atherosclerosis, and their isolated macrophages showed a prevalent pro-inflammatory M1 phenotype. These results further support the hypothesis that individual genetic or epigenetic variation in the expression and activity of the enzymes involved in LC-PUFA metabolism may represent key determinants contributing to explain why a treatment with LC-omega-3 PUFAs may protect some individuals from the risk of inflammatory diseases, including some CVDs, while they do not exert any effects in others.

The inter-individual variability of the genes codifying for enzymes involved in the metabolism of LC-omega-3 PUFAs, and its possible impact on the individual risk for several chronic diseases, has also prompted an investigation into the frequency with which these enzyme variants are present in different populations. Some of these studies have investigated the frequencies of variant alleles of FADS genes in different human populations [55,56,57,58]. Interestingly, the results obtained have allowed us to hypothesize that these enzymes were primary targets of natural selection in some ancestral human populations. This is comprehensible, since LC-PUFAs and their metabolic products represent structural and signaling components essential for biological processes, including the development and functions of neuronal tissues and the regulation of inflammation [59]. In particular, it has been suggested that, due to the large variability in the availability of dietary PUFAs in prehistoric ages, some ancestral populations were forced to adapt to the PUFA scarcity in some environments in order to survive through modifications of their capacity to synthesize and metabolize these fatty acids [60]. For instance, it has been inferred that the pressure for endogenously synthesizing LC-omega-3 PUFAs was reduced in some populations following the emergence of the regular hunting of large animals—excellent sources of these fatty acids [60]. Similarly, it has been hypothesized that the diet of the Inuit population, containing extremely high levels of LC-PUFAs from fish and marine mammals and low levels of linoleic acid, may have caused a pressure to decrease the need to endogenously synthesize LC-omega-3 PUFAs, thus increasing the frequency of specific FADS variants [61,62].

### 4.2. LC-Omega-3 PUFA Forms in Diets or Supplements and Bioavailability

Another possible factor that should not be ignored and that could explain the existing inconsistencies between meta-analyses that systematically examine and compare the outcomes of different human studies on LC-omega-3 PUFAs is the extremely variable bioavailability obtained when different forms of these fatty acids (free fatty acids -FFAs- or esterified fatty acids) are ingested. In fact, the omega-3 PUFA supplements approved by the international health agencies contain these fatty acids in the FFA form or included in ethyl esters, triacylglycerol or monoacylglycerol [63,64,65,66]. Moreover, the bioavailability of omega-3 PUFAs in tissues may also be different depending on the lipid content of a diet and on individual differences in metabolism. For instance, it was previously reported that the bioavailability obtained with triacyl glycerol bound omega-3 PUFAs was higher than that observed with ethyl ester bound forms [67,68]. Moreover, the matrix effect was also analyzed, and it was reported that a sufficient amount of fat in the diet represents an important factor, being able to improve the fatty acid bioavailability up to three times [69]. It was also demonstrated that the galenic form (i.e., microencapsulation, emulsification) can increase up to four times the omega-3 PUFA bioavailability [67,70]. In a recent report from the American Heart association [71], individual metabolic ability was shown to be an additional key factor able to influence the omega-3 PUFA bioavailability. These authors observed that when omega-3 PUFAs were used as lipid-lowering drugs, the best bioavailability was obtained with supplements containing either EPA-FFA or the combination EPA-FFA+DHA-FFA since, differently from omega-3 PUFAs included in triacylglycerol or ethyl esters, they did not need enzymatic hydrolysis to be absorbed.

### 4.3. Omega-3 PUFA Responders and Non-Responders: Reports Applying This Distinction

Based on the inter-individual differences that may exist in response to a supplementation with LC-omega-3 PUFAs, new approaches are starting to be used in designing studies with omega-3 PUFAs, as well as in analyzing the results. In particular, some human studies have used the strategy of sub-dividing the participants into omega-3 PUFA “Rs” and “NRs”. We will critically examine here only the results of those studies that, by applying this strategy, have evaluated the potential of LC-omega-3 PUFAs to reduce hypertriglyceridemia.

#### 4.3.1. Beneficial Effect of Omega-3 PUFAs on Hypertriglyceridemia

Over the last few decades, a great deal of evidence has been accumulated indicating that the most relevant healthy effect of LC-omega-3 PUFAs is the reduction of hypertriglyceridemia. Moreover, we are now witnessing a renewed interest in the potential of these fatty acids to decrease the level of triglycerides (TG), following the conclusive demonstration that hypertriglyceridemia plays a clear pathogenic role in cardiovascular risk [72]. In fact, previously, several epidemiological studies [73,74] had reported the association between high TG serum levels and an increased risk of CVDs. However, a complete agreement on this point was never reached until, more recently, several Mendelian randomization studies were able to shed more light on the direct causal association existing between the levels of TG-rich lipoprotein and the risk of CVDs [72]. This means that, even independently from the level of LDL-C, we now know that hypertriglyceridemia may contribute to the patients’ risk of developing CVDs. With this new scenario, the supplementation with natural dietary components such as omega-3 PUFAs seems to be perceived as a suitable and valuable approach for controlling hypertriglyceridemia in the clinical management of CVD risk. However, the efficiency of these fatty acids in reducing serum TG has not always been observed. Since there is a growing awareness of the different individual capacity of metabolizing and accumulating omega-3 PUFAs in serum and tissues, increasing efforts are being expended to distinguish the subjects that can benefit from dietary supplementations with these fatty acids from those that cannot have any advantage from them. Thus, several reports have distinguished between individuals that positively responded—the “Rs”—and those that did not respond—the “NRs”—to a dietary supplementation with LC-omega-3 PUFAs in the setting of hypertriglyceridemia and related CVDs [75,76,77,78,79]. For instance, in one of the most recent studies analyzed by us, among the 53 statin-treated type 2 diabetic patients enrolled [80], it was possible to identify sub-groups of 10 “Rs” and 10 “NRs”, according to the greater or lesser degree of their response to treatment with LC-omega-3 PUFA-rich fish oil (containing 1.7 g EPA + DHA/day) over 6 weeks. However, in this case, the fish oil treatment was also combined to a supplementation with dark chocolate and green tea (rich in polyphenols and antioxidants, respectively). The inclusion in the “R” or “NR” sub-groups was related to the responses in terms of plasma LDL-C and C-Reactive Protein (hsCRP), respectively, as compared to the control group (receiving soybean oil, regular dark chocolate and anise tea). Interestingly, however, when a substantial reduction in hsCRP (medium decrease, 35%) was observed in the “R” subgroup, it was specifically attributed to the anti-inflammatory activity of the LC-omega-3 PUFAs supplemented. Moreover, it was observed that, among the patients receiving the combined nutritional supplementation, only those having baseline TG values above the median were able to significantly reduce their TG levels. The author hypothesized that this effect could be ascribable specifically to the LC-omega-3 supplemented, since previous data [81] had shown that the extent of the hypotriglyceridemic effect of these fatty acids was strictly related to a high baseline concentration of TG. Only the “R” and “NR” subgroups were enrolled in a second phase of the study, in which the “NR” patients received the control treatment and full statin therapy, while the “R” patients followed a pilot protocol of statin reduction coupled with the combined bioactive nutraceutical supplementation. As noticed above, the study was not designed to give specific information on the effect of omega-3 PUFAs alone. However, it demonstrated that combining a lower statin dose (50% of the full dose) with a dietary supplementation with these fatty acids, polyphenols and antioxidants, produced the same effect as a full statin dose on parameters (such as plasma lipids, inflammatory markers, HDL particles) related to lipidic dysmetabolisms and CVDs. Thus, this therapeutic approach including the identification of “R” patients appears very interesting, and suggests that the identification of specific markers (genetic or metabolic) able to predict the individual responses to omega-3 PUFAs—and to other combined nutraceuticals, as in this particular case—would be essential for their application in the clinical practice. This strategy has been largely suggested in the past several years as an essential step for the applicability of tailored treatments with LC-omega-3 PUFAs in CVD patients. Particularly, several interesting reports published by the Vohl’s group investigated some genetic variants that could be useful in predicting the individual responsiveness to these fatty acids [75,76,77,79]. In a first phase, by using a genoma-wide association study (GWAS) approach, these authors identified the SNPs of 10 loci related to the plasma TG response to an omega-3 PUFA supplementation and computed the associated genetic risk score (GRS) [75,76,77]. Then, more recently [79], they used fine mapping with the aim to compute a novel and more refined GRS and found 31 SNPs associated with the TG response to an omega-3 PUFA supplementation. The higher number of genetic variants identified in the more recent study was able to explain a larger proportion of the TG variance (about 50%) than the 10 SNPs identified previously (about 22%). On this basis, the authors [79] were able to better demonstrate the disparity existing between the “Rs” and “NRs” in terms of the number of at-risk alleles carried. In other terms, this means that the identification of these new genetic variants has the potential to predict, with a higher accuracy, to which category (the “R” or “NR”) each individual belongs.

In a recent sub-study [78] of a previously performed randomized controlled trial, modifications in gene expression and metabolic differences were also identified in individuals that showed a clear TG reduction in response to omega-3 (the “Rs”) with respect to the “NRs”. Among the “Rs”, the reduction in TG was observed not only in plasma, but also in most of the VLDL (four out of six) and HDL subclasses (in three out of four) analyzed in the study. Moreover, by analyzing peripheral blood mononuclear cell gene expression, they found that more than 450 transcripts resulted differentially altered in “Rs”, as compared to “NRs”. By the examination of these transcripts and pathway analysis in the “Rs”, the authors were able to observe alterations in signaling pathways related to development and immune function and lysophosphatidic acid signaling. Interestingly, most of the transcripts differentially altered in the “R” subjects showed a binding site for HNF4-α, which is a transcription factor able to activate the expression of genes involved in lipid metabolism. Its decreased activity was previously related to a decrease in serum TG [82], and this prompted the authors to suggest that an altered activity of this transcription factor could probably be involved in the hypotriglyceridemic effect of these fatty acids.

Furthermore, about twenty years ago, the Minihane’s group [83] already demonstrated that the fasting postprandial TG response to an LC-omega-3 PUFA intervention could be influenced by the individual apolipoprotein E (apoE) genotype in individuals possessing an “atherogenic lipoprotein phenotype” (ALP) that is associated with a high risk for CVDs. In this study, the ALP subjects were treated for six weeks with fish oils corresponding to a supplementation with 3 g/day EPA + DHA and the authors tried to identify which group of patients carrying different genotypes resulted in being the “Best Responders”. The fasting TG levels decreased substantially in all the participants, but those carrying the apoE4 genotype showed the maximal beneficial effect (38% reduction vs. 31% and 35% reduction observed in the apoeE2 and apoE3 subjects, respectively). The hypotriglyceridemic effect at fast was consistent with a large body of evidence that associated it to the inhibitory effect of EPA and DHA on the endogenous very low-density lipoproteins (VLDL) secretion, the inhibition of hepatic lipogenic enzymes, and the induction of apoB degradation [84,85,86]. Of high interest, the authors found that, following the omega-3 PUFA treatment, the postprandial TG increase in response to the meals (TG IAUC) decreased by only 8% if the entire group of participants was considered. However, the fatty acids induced different effects depending on the apoE allele carried by each subject. Whereas only minor changes in TG IAUC (going from 3% to 6%) were observed in individuals carrying the apoE3 or E4 alleles, a 28% reduction in this parameter was observed in the apoE2 subjects. This group also showed a larger omega-3 PUFA-induced increase in LPL activity as compared to the other groups (47% vs. 2% and 17% in apoE3 and apoE4, respectively). Moreover, there was an influence of the genotype also on the undesired increasing effect that these fatty acids may induce on LDLc levels [87,88]. Whereas the LDLc increase was substantial in the apoE4 subjects (16% increase), this effect was much more limited in individuals carrying the apoE2 and apoE3 alleles (3% and 1% increase, respectively). Overall, these results are very interesting and suggest to us that the more suitable candidates for a personalized supplementation with 3 g/day EPA and DHA would be the subjects carrying the apoE2 allele, since, after this intervention, they can substantially reduce both fasting and postprandial TG, with limited adverse consequences on the level of LDLc. However, according to later findings from the same group of authors [89], these hypertriglyceridemic effects seem not to be observed to the same degree if lower LC-omega-3 PUFA doses (0.7–1.8 g/day for 8 weeks) were supplemented. This is consistent with what is reported by different guidelines focusing on LC-omega-3 PUFA dosage and effects released by different health agencies worldwide. They state that omega-3 PUFA doses of about 3–4 g/day are necessary to optimally reduce serum TG [71]. In fact, the lower doses used in the second study [89] were able to induce only a 12% reduction in fasting TG, and this effect resulted to be not significantly related to the genotype, unless the sex was also considered. In fact, only males with an apoE4 genotype showed a doubled capacity to reduce fasting TG (15% and 23% reduction following 8 weeks treatment with 0.7 and 1.8 g/day EPA + DHA) with respect to the whole group. Moreover, for all the genotypes, including the apoE4, the increase in LDLc was only less than one fourth of that observed with the higher omega-3 PUFA dose in the first study (3.6% LDLc increase in the second study vs. 16% increase in the first study). In our opinion, the finding is stimulating, since it indicates that the apoE4 male subjects would represent good candidates for a hypotriglyceridemic treatment with 1.8 g/day omega-3 PUFAs. In fact, only in these subjects this dose could substantially reduce (by more than 20%) the level of TG, having, however, a limited undesirable increasing effect on LDLc (about 3–4% increase).

#### 4.3.2. Beneficial Effect of Omega-3 PUFAs on Other Pathologies

Some studies [90,91,92] that subdivided the participants into omega-3 PUFA “Rs” and “NRs” are also worthy of mention here, even though they were carried out in patients affected by pathologies different from those (dyslipidemias and associated pathologies) specifically analyzed here. In these studies, the participants were affected by metastatic breast cancer [90], depression [91], or ulcerative colitis (UC) [92]. In the first study, Bognoux et al. [90] treated patients affected by metastatic breast cancer with 1.8 g/day DHA both 7–10 days before the initiation of an anthracycline-based chemotherapy (loading period) and during the 5 months of chemotherapy. The authors subdivided the patients in two groups (“high- and low-DHA incorporators”), according to their ability to incorporate DHA after the loading period, considered as the cutoff point for the median value of incorporation. Notably, they found that the outcome of the chemotherapy was substantially improved in the subpopulation of high incorporators, demonstrating for the first time that, among a population of cancer patients treated with omega-3 PUFAs, some can be “Rs” and some can be “NRs” to the treatment itself. Moreover, this finding suggests that the content of DHA in the plasma phospholipids (PL) could reflect the degree of DHA incorporation in normal and tumor tissues and could represent an easily measurable biomarker for establishing the potential response of patients to an omega-3 PUFA treatment. Interestingly, the choice of a DHA loading period lasting about 7–10 days was not casual. In fact, it has been largely demonstrated that the maximum incorporation of LC-omega-3 PUFAs in plasma lipids, and in red blood cells (RBC) or tissues, can be reached after this period, and then stabilizes in a plateau [53]. This suggests that, while we are gaining more knowledge on specific characteristics and biomarkers (genetic- epigenetic- or microbiota-related) to be used in the future, we could now identify the “Rs” by evaluating the levels of LC-omega-3 PUFAs incorporated in PLs or RBCs after less than two weeks of supplementation. Thus, we could quite easily decide which participants in a study are the most eligible to continue a LC-omega-3 PUFA treatment for a longer period, and, thus, presumably obtain more consistent results.

In the second and more recent pilot study [91] that we have considered here, a group of patients showing symptomatic major depressive disorders was supplemented with fish oil (furnishing 1.6 g EPA and 0.8 DHA daily for 6 weeks). The authors considered as “Rs” only the patients (5 patients out of 16) showing a substantial decrease in the score used for evaluating the degree of depression (>50% reduction of the 17-item Hamilton Depression Rating Scale) after the omega-3 PUFA treatment. These patients were considered by the authors to be in remission. In this case, the basal levels of either EPA or DHA in plasma PL was found to be unrelated with the treatment efficacy, and not able to predict the response. However, at the end of the treatment, the “Rs” showed a higher increase of DHA in plasma PLs as compared to the “NRs”, and, particularly, a higher proportion of DHA to EPA in their plasma PLs. There being, however, an amount of EPA twice that of DHA in the fish oil supplemented, what probably differentiated the “Rs” from the “NRs” was the greater ability of the “Rs” to convert EPA into DHA.

In the third study analyzed here [92], the UC patients enrolled were in stable clinical remission and showed high levels (more than 150 μg/g) of fecal calprotectin (FC), a cytosolic protein abundant in neutrophil granulocytes. This fecal protein is considered a useful biomarker able to predict the inflammatory status of the mucosa, the response to therapy, and even anticipate a possible clinical relapse better than other inflammatory biomarkers present in serum [93,94]. In this case, most of the UC patients (17 out of 19) that had received EPA (2 g/day) for three months increased substantially the content of EPA itself and other LC-omega-3 PUFA (DHA and DPA) in their RBCs. Moreover, the treatment was able to reduce significantly and substantially (by more than 60%) the levels of FC in all patients excluding two, where FC remained higher than 150 μg/g even after three months. The authors considered these patients “NRs”, and continued to explore the mechanisms underlying the EPA effects only in the patients that had shown a positive FC response (the “R” patients). Even though the authors did not specify if the same patients who did not respond to the EPA treatment by enhancing their LC-omega-3 PUFA levels in their RBCs were also those that did not reduce their FC levels, this appears highly probable. Therefore, it seems that the same strategy of measuring the incorporation of LC-PUFAs after 7–10 days could be applied also in this pathological condition to easily predict those patients that could potentially be able to respond to the omega-3 PUFA treatment. In fact, only the UC patients showing a high basal level of FC and able to incorporate substantial amounts of LC-omega-3 PUFAs in their RBCs after just 7–10 days of treatment could be considered eligible for continuing this intervention for longer periods. They could be more likely able to suppress colonic mucosa inflammation after the LC-omega-3 PUFA treatment, and to reduce markedly the risk of developing colon cancer.

Based on these findings, we suggest that the same strategy of measuring the incorporation of LC-PUFAs in plasma PL or RBCs after 7–10 days could be applied whenever the potential beneficial effects of LC-PUFA treatments are investigated. This would allow to easily predict which patients could behave as “Rs”, i.e., which could have an advantage from the LC-omega-3 PUFA treatment.

Finally, it is important to highlight the limitations of this review. The main limitation is that the review is a narrative one and is not the result of a complete systematic review process. This implicates the possibility of selection bias, since the methodology adopted in the primary studies analyzed were not examined in a systematic manner. Moreover, the review is limited to the search terms and databases indicated in the “Methodology” section.

## 5. Conclusions

A network of factors (variants of genes involved in PUFA metabolism and the epigenetic regulation of their expression; microbiota profile) are currently being explored, which may influence the individual ability to efficiently respond or not to an omega-3 PUFA treatment. The final goal is the identification of specific factors/biomarkers usable in the clinical practice for an easy pre-identification of those subjects that could benefit from a sustained LC-omega-3 supplementation. This appears particularly interesting in the management of lipid dysmetabolisms and CVDs, where supplementations with these fatty acids are currently indicated. However, while more specific biomarkers are still under study, it is worth considering an approach that has already led to positive results in some human studies. These studies found that the beneficial effects associated with an omega-3 PUFA treatment were mainly observed in those patients (the “Rs”) that were able to incorporate LC-omega-3 PUFAs at the highest levels in their circulating PLs, RBCs or tissues. We suggest here that, after receiving a short LC-omega-3 PUFA treatment, a preliminary check should be performed in all patients, with the aim to identify among the subjects the “best-incorporators” and potential “Rs”, that could be most eligible for long-lasting LC-omega-3 PUFA treatments. This approach would allow us to avoid sustained and sometimes useless treatments in all the patients. Moreover, through this strategy, more homogenous results could be obtained in human studies, and the health potential of these fatty acids could be definitely identified. Importantly, this preliminary check could be performed already after the first 7–10 days of treatment, without any further waste of time, since it has been largely reported that this is the time needed to achieve the maximal incorporation of LC-PUFAs in cells and tissues.

## References

[1] Mason P.R. (2019). New insights into mechanisms of action for omega-3 fatty acids in atherothrombotic cardiovascular disease. Curr. Atheroscler. Rep..

[2] Ajith T.A., Jayakumar T.G. (2019). Omega-3 fatty acids in coronary heart disease: Recent updates and future perspectives. Clin. Exp. Pharm. Physiol..

[3] Saini R.K., Keum Y.S. (2018). Omega-3 and omega-6 polyunsaturated fatty acids: Dietary sources, metabolism, and significance—A review. Life Sci..

[4] Jia X., Kohli P., Virani S.S. (2019). Omega-3 fatty acid and cardiovascular outcomes: Insights from recent clinical trials. Curr. Atheroscler. Rep..

[5] Lenihan-Geels G., Bishop K.S., Ferguson L.R. (2016). Cancer risk and eicosanoid production: Interaction between the protective effect of long chain omega-3 polyunsaturated fatty acid intake and genotype. J. Clin. Med..

[6] Kiyabu G.Y., Inoue M., Saito E., Abe S.K., Sawada N., Ishihara J., Iwasaki M., Yamaji T., Shimazu T., Sasazuki S. (2015). Fish, n-3 polyunsaturated fatty acids and n-6 polyunsaturated fatty acids intake and breast cancer risk: The Japan Public Health Center-based prospective study. Int. J. Cancer.

[7] Nabavi S.F., Bilotto S., Russo G.L., Orhan I.E., Habtemariam S., Daglia M., Devi K.P., Loizzo M.R., Tundis R., Nabavi S.M. (2015). Omega-3 polyunsaturated fatty acids and cancer: Lessons learned from clinical trials. Cancer Metastasis Rev..

[8] Chilton F.H., Dutta R., Reynolds L.M., Sergeant S., Mathias R.A., Seeds M.C. (2017). Precision nutrition and omega-3 polyunsaturated fatty acids: A case for personalized supplementation approaches for the prevention and management of human diseases. Nutrients.

[9] Ooi E.M., Watts G.F., Ng T.W., Barrett P.H. (2015). Effect of dietary fatty acids on human lipoprotein metabolism: A comprehensive update. Nutrients.

[10] Minihane A.M. (2016). Impact of genotype on EPA and DHA status and responsiveness to increased intakes. Nutrients.

[11] Brayner B., Kaur G., Keske M.A., Livingstone K.M. (2018). FADS Polymorphism, Omega-3 Fatty Acids and Diabetes Risk: A Systematic Review. Nutrients.

[12] Ramos-Lopez O., Milagro F.I., Allayee H., Chmurzynska A., Choi M.S., Curi R., De Caterina R., Ferguson L.R., Goni L., Kang J.X. (2017). Guide for current nutrigenetic, nutrigenomic, and nutriepigenetic approaches for precision nutrition involving the prevention and management of chronic diseases associated with obesity. J. Nutr. Nutr..

[13] Sioen I., van Lieshout L., Eilander A., Fleith M., Lohner S., Szommer A., Petisca C., Eussen S., Forsyth S., Calder P.C. (2017). Systematic review on n-3 and n-6 polyunsaturated fatty acid intake in european countries in light of the current recommendations—Focus on specific population groups. Ann. Nutr. Metab..

[14] Patel J.V., Tracey I., Hughes E.A., Lip G.Y. (2010). Omega-3 polyunsaturated acids and cardiovascular disease: Notable ethnic differences or unfulfilled promise?. J. Thromb. Haemost..

[15] Tang X., Li Z.J., Xu J., Xue Y., Li J.Z., Wang J.F., Yanagita T., Xue C.H., Wang Y.M. (2012). Short term effects of different omega-3 fatty acid formulation on lipid metabolism in mice fed high or low fat diet. Lipids Health Dis..

[16] Sun D., Zhang L., Chen H., Feng R., Cao P., Liu Y. (2017). Effects of Antarctic krill oil on lipid and glucose metabolism in C57BL/6J mice fed with high fat diet. Lipids Health Dis..

[17] Chacińska M., Zabielski P., Książek M., Szałaj P., Jarząbek K., Kojta I., Chabowski A., Błachnio-Zabielska A.U. (2019). The Impact of OMEGA-3 Fatty acids supplementation on insulin resistance and content of adipocytokines and biologically active lipids in adipose tissue of high-fat diet fed rats. Nutrients.

[18] Sauvageot N., Alkerwi A., Albert A., Guillaume M. (2013). Use of food frequency questionnaire to assess relationships between dietary habits and cardiovascular risk factors in NESCAV study: Validation with biomarkers. Nutr. J..

[19] Von Schacky C. (2014). Omega-3 index and cardiovascular health. Nutrients.

[20] Zárate R., El Jaber-Vazdekis N., Tejera N., Pérez J.A., Rodríguez C. (2017). Significance of long chain polyunsaturated fatty acids in human health. Clin. Transl. Med..

[21] Simopoulos A.P. (2016). An increase in the omega-6/omega-3 fatty acid ratio increases the risk for obesity. Nutrients.

[22] Welch A.A., Shakya-Shrestha S., Lentjes M.A., Wareham N.J., Khaw K.T. (2010). Dietary intake and status of n-3 polyunsaturated fatty acids in a population of fish-eating and non-fish-eating meat-eaters, vegetarians, and vegans and the precursor-product ratio of a-linolenic acid to longchain n-3 polyunsaturated fatty acids: Results from the EPIC-Norfolk cohort. Am. J. Clin. Nutr..

[23] Jernerén F., Cederholm T., Refsum H., Smith A.D., Turner C., Palmblad J., Eriksdotter M., Hjorth E., Faxen-Irving G., Wahlund L.O. (2019). Homocysteine status modifies the treatment effect of omega-3 fatty acids on cognition in a randomized clinical trial in mild to moderate Alzheimer’s disease: The OmegAD study. J. Alzheimers Dis..

[24] Marchesi J.R., Adams D.H., Fava F., Hermes G.D., Hirschfield G.M., Hold G., Quraishi M.N., Kinross J., Smidt H., Tuohy K.M. (2016). The gut microbiota and host health: A new clinical frontier. Gut.

[25] Lazar V., Ditu L.M., Pircalabioru G.G., Gheorghe I., Curutiu C., Holban A.M., Picu A., Petcu L., Chifiriuc M.C. (2018). Aspects of gut microbiota and immune system interactions in infectious diseases, immunopathology, and cancer. Front. Immunol..

[26] Clemente J.C., Ursell L.K., Parfrey L.W., Knight R. (2012). The impact of the gut microbiota on human health: An integrative view. Cell.

[27] Human Microbiome Project Consortium (2012). Structure, function and diversity of the healthy human microbiome. Nature.

[28] Costantini L., Molinari R., Farinon B., Merendino N. (2017). Impact of omega-3 fatty acids on the gut microbiota. Int. J. Mol. Sci..

[29] Bellenger J., Bellenger S., Escoula Q., Bidu C., Narce M. (2019). N-3 polyunsaturated fatty acids: An innovative strategy against obesity and related metabolic disorders, intestinal alteration and gut microbiota dysbiosis. Biochimie.

[30] Kaliannan K., Wang B., Li X.Y., Bhan A.K., Kang J.X. (2016). Omega-3 fatty acids prevent early-life antibiotic exposure-induced gut microbiota dysbiosis and later-life obesity. Int. J. Obes..

[31] Gui L., Chen S., Wang H., Ruan M., Liu Y., Li N., Zhang H., Liu Z. (2019). ω-3 PUFAs alleviate high-fat diet-induced circadian intestinal microbes dysbiosis. Mol. Nutr. Food Res..

[32] Org E., Parks B.W., Joo J.W., Emert B., Schwartzman W., Kang E.Y., Mehrabian M., Pan C., Knight R., Gunsalus R. (2015). Genetic and environmental control of host-gut microbiota interactions. Genome Res..

[33] Cao Z.J., Yu J.C., Kang W.M., Ma Z.Q., Ye X., Tian S.B. (2014). Effect of n-3 polyunsaturated fatty acids on gut microbiota and endotoxin levels in portal vein of rats fed with high-fat diet. Acta Acad. Med. Sin..

[34] Djuric Z., Bassis C.M., Plegue M.A., Sen A., Turgeon D.K., Herman K., Young V.B., Brenner D.E., Ruffin M.T. (2019). Increases in colonic bacterial diversity after ω-3 fatty acid supplementation predict decreased colonic prostaglandin E2 concentrations in healthy adults. J. Nutr..

[35] Dubois R.N. (2014). Role of inflammation and inflammatory mediators in colorectal cancer. Trans. Am. Clin. Climatol. Assoc..

[36] Ley R.E. (2016). Gut microbiota in 2015: Prevotella in the gut: Choose carefully. Nat. Rev. Gastroenterol. Hepatol..

[37] Roager H.M., Licht T.R., Poulsen S.K., Larsen T.M., Bahl M.I. (2014). Microbial enterotypes, inferred by the prevotella-to-bacteroides ratio, remained stable during a 6-month randomized controlled diet intervention with the new nordic diet. Appl. Environ. Microbiol..

[38] Tanaka T., Shen J., Abecasis G.R., Kisialiou A., Ordovas J.M., Guralnik J.M., Singleton A., Bandinelli S., Cherubini A., Arnett D. (2009). Genome-wide association study of plasma polyunsaturated fatty acids in the InCHIANTI Study. PLoS Genet..

[39] O’Neill C.M., Minihane A.M. (2017). The impact of fatty acid desaturase genotype on fatty acid status and cardiovascular health in adults. Proc. Nutr. Soc..

[40] Briscoe C.P., Tadayyon M., Andrews J.L., Benson W.G., Chambers J.K., Eilert M.M., Ellis C., Elshourbagy N.A., Goetz A.S., Minnick D.T. (2003). The orphan g protein-coupled receptor gpr40 is activated by medium and long chain fatty acids. J. Biol. Chem..

[41] Oh D.Y., Talukdar S., Bae E.J., Imamura T., Morinaga H., Fan W., Li P., Lu W.J., Watkins S.M., Olefsky J.M. (2010). Gpr120 is an omega-3 fatty acid receptor mediating potent anti-inflammatory and insulin-sensitizing effects. Cell.

[42] Shaikh S.R. (2012). Biophysical and biochemical mechanisms by which dietary N-3 polyunsaturated fatty acids from fish oil disrupt membrane lipid rafts. J. Nutr. Biochem..

[43] Turk H.F., Chapkin R.S. (2013). Membrane lipid raft organization is uniquely modified by n-3 polyunsaturated fatty acids. Prostaglandins Leukot. Essent. Fatty Acids.

[44] Serhan C.N. (2014). Pro-resolving lipid mediators are leads for resolution physiology. Nature.

[45] Serhan C.N., Levy B.D. (2018). Resolvins in inflammation: Emergence of the pro-resolving superfamily of mediators. J. Clin. Investig..

[46] Reynolds L.M., Howard T.D., Ruczinski I., Kanchan K., Seeds M.C., Mathias R.A., Chilton F.H. (2018). Tissue-specific impact of FADS cluster variants on FADS1 and FADS2 gene expression. PLoS ONE.

[47] Lupu D.S., Cheatham C.L., Corbin K.D., Niculescu M.D. (2015). Genetic and epigenetic transgenerational implications related to omega-3 fatty acids. Part I: Maternal FADS2 genotype and DNA methylation correlate with polyunsaturated fatty acid status in toddlers: An exploratory analysis. Nutr. Res..

[48] Howard T.D., Mathias R.A., Seeds M.C., Herrington D.M., Hixson J.E., Shimmin L.C., Hawkins G.A., Sellers M., Ainsworth H.C., Sergeant S. (2014). DNA methylation in an enhancer region of the FADS cluster is associated with fads activity in human liver. PLoS ONE.

[49] He Z., Zhang R., Jiang F., Zhang H., Zhao A., Xu B., Jin L., Wang T., Jia W., Jia W. (2018). *FADS1-FADS2* genetic polymorphisms are associated with fatty acid metabolism through changes in DNA methylation and gene expression. Clin. Epigenetics.

[50] Ajith T.A. (2018). A recent update on the effects of omega-3 fatty acids in Alzheimer’s Disease. Curr. Clin. Pharmacol..

[51] Talamonti E., Pauter A.M., Asadi A., Fischer A.W., Chiurchiù V., Jacobsson A. (2017). Impairment of systemic DHA synthesis affects macrophage plasticity and polarization: Implications for DHA supplementation during inflammation. Cell. Mol. Life Sci..

[52] Yang B., Fritsche K.L., Beversdorf D.Q., Gu Z., Lee J.C., Folk W.R., Greenlief C.M., Sun G.Y. (2019). Yin-Yang mechanisms regulating peroxidation of docosahexaenoic acid and arachidonic acid in the central nervous system. Front. Neurol..

[53] Fasano E., Serini S., Cittadini A., Calviello G. (2017). Long-chain n-3 PUFA against breast and prostate cancer: Which are the appropriate doses for intervention studies in animals and humans?. Crit. Rev. Food Sci. Nutr..

[54] Gromovsky A.D., Schugar R.C., Brown A.L., Helsley R.N., Burrows A.C., Ferguson D., Zhang R., Sansbury B.E., Lee R.G., Morton R.E. (2018). Δ-5 Fatty acid desaturase *FADS1* impacts metabolic disease by balancing proinflammatory and proresolving lipid mediators. Arterioscler. Thromb. Vasc. Biol..

[55] Buckley M.T., Racimo F., Allentoft M.E., Jensen M.K., Jonsson A., Huang H., Hormozdiari F., Sikora M., Marnetto D., Eskin E. (2017). Selection in europeans on fatty acid desaturases associated with dietary changes. Mol. Biol. Evol..

[56] Harris D.N., Ruczinski I., Yanek L.R., Becker L.C., Becker D.M., Guio H., Cui T., Chilton F.H., Mathias R.A., O’Connor T.D. (2019). Evolution of Hominin Polyunsaturated Fatty Acid Metabolism: From Africa to the New World. Genome Biol. Evol..

[57] Mathias R.A., Sergeant S., Ruczinski I., Torgerson D.G., Hugenschmidt C.E., Kubala M., Vaidya D., Suktitipat B., Ziegler J.T., Ivester P. (2011). The impact of FADS genetic variants on ω6 polyunsaturated fatty acid metabolism in African Americans. BMC Genet..

[58] Ameur A., Enroth S., Johansson A., Zaboli G., Igl W., Johansson A.C., Rivas M.A., Daly M.J., Schmitz G., Hicks A.A. (2012). Genetic adaptation of fatty-acid metabolism: A human-specific haplotype increasing the biosynthesis of long-chain omega-3 and omega-6 fatty acids. Am. J. Hum. Genet..

[59] Serini S., Calviello G. (2016). Reduction of oxidative/nitrosative stress in brain and its involvement in the neuroprotective effect of n-3 PUFA in Alzheimer’s Disease. Curr. Alzheimer Res..

[60] Mathias R.A., Fu W., Akey J.M., Ainsworth H.C., Torgerson D.G., Ruczinski I., Sergeant S., Barnes K.C., Chilton F.H. (2012). Adaptive evolution of the FADS gene cluster within Africa. PLoS ONE.

[61] Fumagalli M., Moltke I., Grarup N., Racimo F., Bjerregaard P., Jørgensen M.E., Korneliussen T.S., Gerbault P., Skotte L., Linneberg A. (2015). Greenlandic Inuit show genetic signatures of diet and climate adaptation. Science.

[62] Amorim C.E., Nunes K., Meyer D., Comas D., Bortolini M.C., Salzano F.M., Hünemeier T. (2017). Genetic signature of natural selection in first Americans. Proc. Natl. Acad. Sci. USA.

[63] Davidson M.H., Stein E.A., Bays H.E., Maki K.C., Doyle R.T., Shalwitz R.A., Ballantyne C.M., Ginsberg H.N. (2007). COMBination of prescription Omega-3 with Simvastatin (COMBOS) Investigators. Efficacy and tolerability of adding prescription omega-3 fatty acids 4 g/d to simvastatin 40 mg/d in hypertriglyceridemic patients: An 8-week, randomized, double-blind, placebo-controlled study. Clin. Ther..

[64] Bays H.E., Tighe A.P., Sadovsky R., Davidson M.H. (2008). Prescription omega-3 fatty acids and their lipid effects: Physiologic mechanisms of action and clinical implications. Expert Rev. Cardiovasc. Ther..

[65] Cruz-Hernandez C., Thakkar S.K., Moulin J., Oliveira M., Masserey-Elmelegy I., Dionisi F., Destaillats F. (2012). Benefits of structured and free monoacylglycerols to deliver eicosapentaenoic (EPA) in a model of lipid malabsorption. Nutrients.

[66] Cruz-Hernandez C., Destaillats F., Thakkar S.K., Goulet L., Wynn E., Grathwohl D., Roessle C., de Giorgi S., Tappy L., Giuffrida F. (2016). Monoacylglycerol-enriched oil increases EPA/DHA delivery to circulatory system in humans with induced lipid malabsorption conditions. J. Lipid Res..

[67] Schuchardt J.P., Hahn A. (2013). Bioavailability of long-chain omega-3 fatty acids. Prostaglandins Leukot. Essent. Fatty Acids.

[68] West A.L., Burdge G.C., Calder P.C. (2016). Lipid structure does not modify incorporation of EPA and DHA into blood lipids in healthy adults: A randomised-controlled trial. Br. J. Nutr..

[69] Lawson L.D., Hughes B.G. (1988). Absorption of eicosapentaenoic acid and docosahexaenoic acid from fish oil triacylglycerols or fish oil ethyl esters co-ingested with a high-fat meal. Biochem. Biophys. Res. Commun..

[70] Garaiova I., Guschina I.A., Plummer S.F., Tang J., Wang D., Plummer N.T. (2007). A randomised cross-over trial in healthy adults indicating improved absorption of omega-3 fatty acids by pre-emulsification. Nutr. J..

[71] Skulas-Ray A.C., Wilson P.W.F., Harris W.S., Brinton E.A., Kris-Etherton P.M., Richter C.K., Jacobson T.A., Engler M.B., Miller M., Robinson J.G. (2019). Omega-3 fatty acids for the management of hypertriglyceridemia: A science advisory from the American Heart Association. Circulation.

[72] Arca M., Borghi C., Pontremoli R., De Ferrari G.M., Colivicchi F., Desideri G., Temporelli P.L. (2018). Hypertriglyceridemia and omega-3 fatty acids: Their often overlooked role in cardiovascular disease prevention. Nutr. Metab. Cardiovasc. Dis..

[73] Hokanson J.E., Austin M.A. (1996). Plasma triglyceride level is a risk factor for cardiovascular disease independent of high-density lipoprotein cholesterol level: A meta-analysis of population-based prospective studies. J. Cardiovasc. Risk.

[74] Sarwar N., Danesh J., Eiriksdottir G., Sigurdsson G., Wareham N., Bingham S., Boekholdt S.M., Khaw K.T., Gudnason V. (2007). Triglycerides and the risk of coronary heart disease: 10,158 incident cases among 262,525 participants in 29 Western prospective studies. Circulation.

[75] Rudkowska I., Paradis A.M., Thifault E., Julien P., Barbier O., Couture P., Lemieux S., Vohl M.C. (2013). Differences in metabolomic and transcriptomic profiles between responders and non-responders to an n-3 polyunsaturated fatty acids (PUFAs) supplementation. Genes Nutr..

[76] Rudkowska I., Julien P., Couture P., Lemieux S., Tchernof A., Barbier O., Vohl M.C. (2014). Cardiometabolic risk factors are influenced by Stearoyl-CoA Desaturase (SCD)-1 gene polymorphisms and n-3 polyunsaturated fatty acid supplementation. Mol. Nutr. Food Res..

[77] Rudkowska I., Guénard F., Julien P., Couture P., Lemieux S., Barbier O., Calder P.C., Minihane A.M., Vohl M.C. (2014). Genome-wide association study of the plasma triglyceride response to an n-3 polyunsaturated fatty acid supplementation. J. Lipid Res..

[78] Rundblad A., Larsen S.V., Myhrstad M.C., Ottestad I., Thoresen M., Holven K.B., Ulven S.M. (2019). Differences in peripheral blood mononuclear cell gene expression and triglyceride composition in lipoprotein subclasses in plasma triglyceride responders and non-responders to omega-3 supplementation. Genes Nutr..

[79] Vallée Marcotte B., Guénard F., Lemieux S., Couture P., Rudkowska I., Calder P.C., Minihane A.M., Vohl M.C. (2019). Fine mapping of genome-wide association study signals to identify genetic markers of the plasma triglyceride response to an omega-3 fatty acid supplementation. Am. J. Clin. Nutr..

[80] Scolaro B., Nogueira M.S., Paiva A., Bertolami A., Barroso L.P., Vaisar T., Heffron S.P., Fisher E.A., Castro I.A. (2018). Statin dose reduction with complementary diet therapy: A pilot study of personalized medicine. Mol. Metab..

[81] Jacobson T.A. (2008). Role of n-3 fatty acids in the treatment of hypertriglyceridemia and cardiovascular disease. Am. J. Clin. Nutr..

[82] Hayhurst G.P., Lee Y.H., Lambert G., Ward J.M., Gonzalez F.J. (2001). Hepatocyte nuclear factor 4alpha (nuclear receptor 2A1) is essential for maintenance of hepatic gene expression and lipid homeostasis. Mol. Cell. Biol..

[83] Minihane A.M., Khan S., Leigh-Firbank E.C., Talmud P., Wright J.W., Murphy M.C., Griffin B.A., Williams C.M. (2000). ApoE polymorphism and fish oil supplementation in subjects with an atherogenic lipoprotein phenotype. Arterioscler. Thromb. Vasc. Biol..

[84] Harris W.S. (1989). Fish oils and plasma lipid and lipoprotein metabolism in humans: A critical review. J. Lipid Res..

[85] Nestel P.J., Connor W.E., Reardon M.R., Connor S., Wong S., Boston R. (1984). Suppression by diets rich in fish oil of very low density lipoprotein production in man. J. Clin. Investig..

[86] Kendrick J.S., Higgins J.A. (1999). Dietary fish oils inhibit early events in the assembly of very low density lipoproteins and target apoB for degradation within the rough endoplasmic reticulum of hamster hepatocytes. J. Lipid Res..

[87] Jacobson T.A., Glickstein S.B., Rowe J.D., Soni P.N. (2012). Effects of eicosapentaenoic acid and docosahexaenoic acid on low-density lipoprotein cholesterol and other lipids: A review. J. Clin. Lipidol..

[88] Yang Z.H., Amar M., Sampson M., Courville A.B., Sorokin A.V., Gordon S.M., Aponte A.M., Stagliano M., Playford M.P., Fu Y.P. (2020). Comparison of omega-3 eicosapentaenoic acid versus docosahexaenoic acid-rich fish oil supplementation on plasma lipids and lipoproteins in normolipidemic adults. Nutrients.

[89] Caslake M.J., Miles E.A., Kofler B.M., Lietz G., Curtis P., Armah C.K., Kimber A.C., Grew J.P., Farrell L., Stannard J. (2008). Effect of sex and genotype on cardiovascular biomarker response to fish oils: The FINGEN Study. Am. J. Clin. Nutr..

[90] Bougnoux P., Hajjaji N., Ferrasson M.N., Giraudeau B., Couet C., Le Floch O. (2009). Improving outcome of chemotherapy of metastatic breast cancer by docosahexaenoic acid: A phase II trial. Br. J. Cancer.

[91] Ganança L., Galfalvy H.C., Oquendo M.A., Hezghia A., Cooper T.B., Mann J.J., Sublette M.E. (2017). Lipid correlates of antidepressant response to omega-3 polyunsaturated fatty acid supplementation: A pilot study. Prostaglandins Leukot. Essent. Fatty Acids.

[92] Prossomariti A., Scaioli E., Piazzi G., Fazio C., Bellanova M., Biagi E., Candela M., Brigidi P., Consolandi C., Balbi T. (2017). Short-term treatment with eicosapentaenoic acid improves inflammation and affects colonic differentiation markers and microbiota in patients with ulcerative colitis. Sci. Rep..

[93] Poullis A., Foster R., Mendall M.A., Fagerhol M.K. (2003). Emerging role of calprotectin in gastroenterology. J. Gastroenterol. Hepatol..

[94] Mooiweer E., Fidder H.H., Siersema P.D., Laheij R.J., Oldenburg B. (2014). Fecal calprotectin testing can identify ineffective colorectal cancer surveillance procedures in patients with longstanding colitis. Inflamm. Bowel Dis..

